# Inflammasome-mediated cell death in response to bacterial pathogens that access the host cell cytosol: lessons from *legionella pneumophila*

**DOI:** 10.3389/fcimb.2013.00111

**Published:** 2013-12-27

**Authors:** Cierra N. Casson, Sunny Shin

**Affiliations:** Department of Microbiology, Perelman School of Medicine, University of PennsylvaniaPhiladelphia, PA, USA

**Keywords:** *Legionella pneumophila*, inflammasome, cell death, pyroptosis, caspase-11, caspase-1

## Abstract

Cell death can be critical for host defense against intracellular pathogens because it eliminates a crucial replicative niche, and pro-inflammatory cell death can alert neighboring cells to the presence of pathogenic organisms and enhance downstream immune responses. Pyroptosis is a pro-inflammatory form of cell death triggered by the inflammasome, a multi-protein complex that assembles in the cytosol to activate caspase-1. Inflammasome activation by pathogens hinges upon violation of the host cell cytosol by activities such as the use of pore-forming toxins, the use of specialized secretion systems, or the cytosolic presence of the pathogen itself. Recently, a non-canonical inflammasome has been described that activates caspase-11 and also leads to pro-inflammatory cell death. Caspase-11 is activated rapidly and robustly in response to violation of the cytosol by bacterial pathogens as well. In this mini-review, we describe the canonical and non-canonical inflammasome pathways that are critical for host defense against a model intracellular bacterial pathogen that accesses the host cytosol—*Legionella pneumophila*.

## Introduction

Cell death is an important innate immune effector mechanism to aid in clearance of intracellular pathogens, as it can eliminate a pathogen's replicative niche. Additionally, pro-inflammatory cell death can be critical for alerting neighboring cells to the presence of invading pathogens (Kono and Rock, 2008; Bergsbaken et al., 2009). The pro-inflammatory form of cell death known as pyroptosis is critical both for clearance of bacterial pathogens and for release of important proinflammatory cytokines *in vivo* (Fink and Cookson, 2005; Miao et al., 2010a). Pyroptosis is initiated in response to violation of the host cell cytosol by pathogenic microbes (Lamkanfi and Dixit, 2009). Violation of the cytosol can occur either by access via bacterial secretion systems, such as type IV (T4SS) or type III (T3SS) secretion systems, or by physical entry of a pathogen into the cytosol. Here, we discuss how cells induce proinflammatory cell death in response to microbes gaining cytosolic access by using *Legionella pneumophila* as a model intracellular pathogen.

## NOD-like receptors respond to cytosolic access by pathogens

Pattern recognition receptors (PRRs) are critical initiators of host defense against invading microorganisms (Janeway and Medzhitov, 2002; Medzhitov, 2007). Surface and endosomally-associated PRRs, such as Toll-like receptors (TLRs), recognize pathogen-associated molecular patterns found in the extracellular space (Janeway and Medzhitov, 2002). However, many pathogenic organisms have mechanisms for accessing the host cytosol. Thus, many cells encode cytosolic PRRs, such as nucleotide-binding oligomerization domain (NOD)-like receptors (NLRs) (Harton et al., 2002), which act as guardians of cytosolic sanctity (Lamkanfi and Dixit, 2009). NLRs respond to “patterns of pathogenesis,” such as membrane disruption, delivery of bacterial molecules into the host cytosol via specialized secretion systems, or pore-forming toxins, by activating the inflammasome (Fritz et al., 2006; Lamkanfi and Dixit, 2009; Vance et al., 2009; Davis et al., 2011; Franchi et al., 2012).

## Caspase-1-dependent inflammasomes

The canonical inflammasome is a multi-protein complex that assembles in the cytosol to activate the enzyme caspase-1, also known as interleukin-1β (IL-1β)-converting enzyme (ICE) (Martinon et al., 2002). Caspase-1 regulates secretion of IL-1 family cytokines and a pro-inflammatory form of cell death termed pyroptosis (Rathinam et al., 2012a). Caspase-1 processes IL-1β and IL-18 into their mature forms and aids in their secretion (Howard et al., 1991; Thornberry et al., 1992; Ghayur et al., 1997; Gu et al., 1997). Caspase-1 does not cleave IL-1α, though it can aid in IL-1α secretion as well (Keller et al., 2008). IL-1 family cytokines act *in vivo* to enhance immune responses against invading microorganisms (Labow et al., 1997; Bohn et al., 1998; Dinarello, 2009). Additionally, caspase-1-mediated pyroptosis enhances clearance of bacterial pathogens *in vivo* (Miao et al., 2010a).

NLRs respond to different stimuli when activating the inflammasome. Few NLRs have been shown to bind directly to their implicated substrates, and some are activated by a wide variety of stimuli. For example, NLRP3 responds to stimuli ranging from bacterial RNA to extracellular adenosine triphosphate and uric acid crystals (Kanneganti et al., 2006; Mariathasan et al., 2006; Martinon et al., 2006). Absent in melanoma 2 (AIM2) responds to the presence of cytosolic double-stranded DNA (Hornung et al., 2009; Roberts et al., 2009). In mice, ICE-protease activating factor (IPAF)/NLR family, CARD domain containing 4 (NLRC4) mediates inflammasome activation in response to three distinct stimuli—flagellin, the conserved inner rod component of the bacterial T3SS (PrgJ), and T3SS needle proteins (Franchi et al., 2006; Miao et al., 2006, 2010b; Lightfield et al., 2011; Yang et al., 2013). Biochemical studies have shown that the NLRs neuronal apoptosis inhibitory protein 5 (NAIP5) and NAIP6 co-immunoprecipitate with flagellin, while NAIP2 interacts specifically with PrgJ and NAIP1 interacts with the needle proteins (Kofoed and Vance, 2011; Zhao et al., 2011; Yang et al., 2013). NLRC4 appears to be an important adaptor for the NAIP receptors. The adaptor protein apoptosis-associated speck-like protein containing a carboxy-terminal caspase recruitment domain (ASC) often bridges the interaction between NLRs and caspase-1, allowing for oligomerization and auto-processing of caspase-1 for activation (Srinivasula et al., 2002). Caspase-1 auto-processing is required for cytokine cleavage and secretion, though cell death can occur independently of caspase-1 proteolysis (Broz et al., 2010).

## The non-canonical inflammasome

Experiments examining inflammasome activation were first performed with macrophages from mice that lack caspase-1, and it was concluded that caspase-1 is solely responsible for inflammasome activation. However, the strain of mice used to generate the original caspase-1 knockout has a caspase-11 polymorphism that eliminates protein expression. Thus, the original mice lack both caspase-1 and caspase-11 (Kuida et al., 1995; Kayagaki et al., 2011). Though it was reported that caspase-11 mediates septic shock *in vivo*, the cell-intrinsic role of caspase-11 in response to bacterial pathogens remained unclear (Wang et al., 1996, 1998). Recently, however, a non-canonical caspase-11-dependent inflammasome has been described that contributes to IL-1α, IL-1β, and IL-18 secretion and cell death in response to many Gram-negative bacteria. Caspase-11 is activated with delayed kinetics, taking 16–24 h *in vitro*, in response to bacteria that do not typically access the host cell cytosol, such as non-pathogenic *Escherichia coli* (Kayagaki et al., 2011). For many Gram-negative bacteria, non-canonical inflammasome activation requires TIR-domain-containing adaptor-inducing interferon-β (TRIF) and type I interferon (IFN) signaling downstream of TLR4 (Broz et al., 2012; Gurung et al., 2012; Rathinam et al., 2012b). Additionally, cytosolic lipopolysaccharide (LPS) activates caspase-11 independently of TLR4 (Hagar et al., 2013; Kayagaki et al., 2013). Pathogens that access or enter the host cytosol also induce non-canonical inflammasome activation, and this activation is more rapid than for other Gram-negative bacteria. One robust activator of the non-canonical inflammasome is the intracellular pathogen *Legionella pneumophila* (Aachoui et al., 2013; Case et al., 2013; Casson et al., 2013).

## Legionella pneumophila

*L. pneumophila* is a Gram-negative bacterium that causes the severe pneumonia Legionnaires' disease (Fraser et al., 1977; McDade et al., 1977). *L. pneumophila* uses its *dot*/*icm*-encoded T4SS to translocate effector proteins into the host cytosol to establish an endoplasmic reticulum-derived vacuole that supports bacterial replication (Marra et al., 1992; Berger and Isberg, 1993; Sadosky et al., 1993; Roy et al., 1998; Segal et al., 1998; Vogel et al., 1998). The natural host for *L. pneumophila* is amoebae in aquatic reservoirs (Rowbotham, 1980; Fliermans et al., 1981), so while it has evolved to evade amoebic host defenses, it is not thought to have evolved to evade mammalian-specific immune responses. Therefore, as a consequence of accessing the host cytosol in mammalian cells, *L. pneumophila* triggers multiple pathways that elicit cell-intrinsic immune responses and induce cell death. These robust immune responses make the bacterium valuable for studying host defense against intracellular pathogens.

## *L. pneumophila* and caspase-1-dependent inflammasome activation

**Figure 1:**
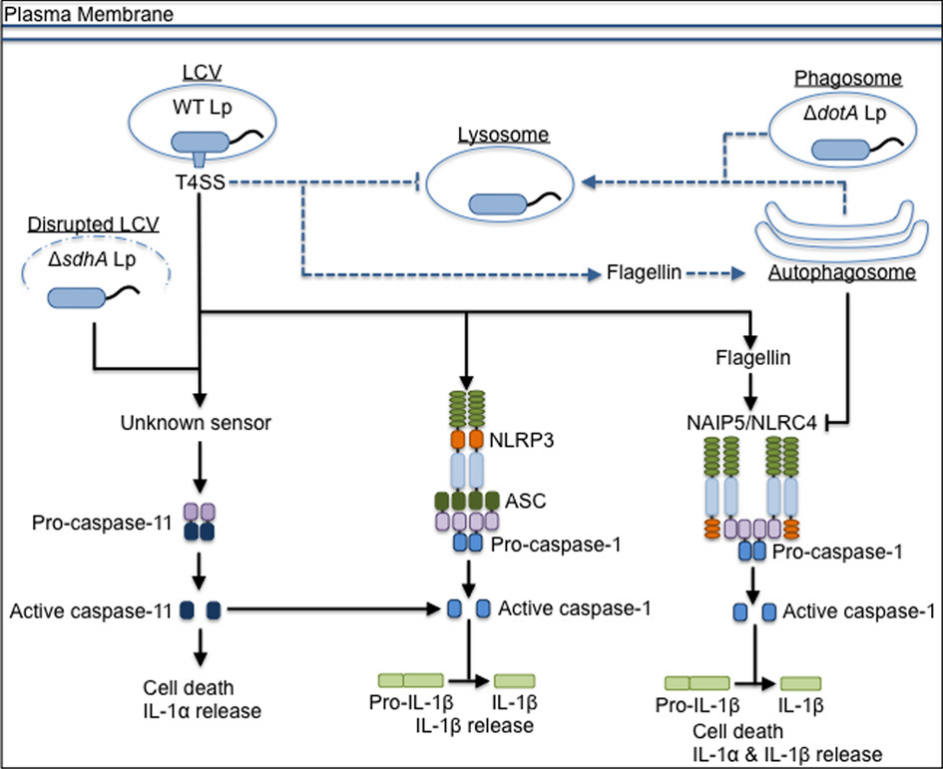
**The inflammasome-mediated response to *L. pneumophila* in murine macrophages**. *L. pneumophila* that do not express a functional T4SS (Δ*dotA* Lp) traffic to the lysosome, but wild-type *L. pneumophila* (WT Lp) use the T4SS to translocate effectors into the host cytosol to establish a replicative niche, the *Legionella*-containing vacuole (LCV), and block fusion with lysosomes. WT Lp triggers canonical caspase-1-dependent inflammasome activation through detection of translocated flagellin by NAIP5/NLRC4. NLRP3 and ASC contribute to IL-1β secretion in response to WT Lp. Detection of T4SS activity through an unknown sensor leads to caspase-11 activation, which contributes to inflammasome activation. Caspase-11 also responds to bacteria that aberrantly enter the cytosol (Δ*sdhA* Lp) due to loss of LCV membrane integrity. Translocated flagellin triggers trafficking of WT Lp to the autophagosome, and induction of autophagy negatively regulates pyroptosis if there are low levels of flagellin in the host cell cytosol. Dashed lines represent vesicular trafficking patterns. Solid lines represent pathways for activation of the host response. Arrows at the end of lines represent induction, while flat bars at the end of lines represent inhibition.

It is well-understood that *L. pneumophila* triggers inflammasome activation and pyroptosis as a consequence of flagellin expression and T4SS activity (Figure 1). In murine macrophages, detection of flagellin by BIRC1e/NAIP5 mediates pyroptosis and contributes to restriction of *L. pneumophila* replication both *in vitro* and *in vivo* (Growney and Dietrich, 2000; Diez et al., 2003; Wright et al., 2003; Derré and Isberg, 2004; Zamboni et al., 2006; Kofoed and Vance, 2011; Zhao et al., 2011). Flagellin-deficient *L. pneumophila* (Δ*flaA* Lp) evade NAIP5-mediated restriction and replicate in NAIP5-sufficient macrophages from C57BL/6 (B6) mice, in part because they do not induce as much caspase-1-dependent cell death as wild-type (WT) Lp (Molofsky et al., 2006; Ren et al., 2006). NLRC4 also acts upstream of caspase-1 to induce flagellin-mediated restriction of replication, pore formation in the host membrane, and IL-1β release (Zamboni et al., 2006; Silveira and Zamboni, 2010). NLRC4 co-immunoprecipitates with NAIP5, consistent with the model that NLRC4 is an adaptor for NAIP5 (Zamboni et al., 2006; Kofoed and Vance, 2011; Zhao et al., 2011). The NAIP5/NLRC4-dependent cell death induced in B6 macrophages requires cytosolic access, as T4SS-deficient mutants (Δ*dotA* Lp) do not activate the inflammasome. These data suggest that flagellin is translocated through the T4SS into the host cytosol during infection, though this has not been shown experimentally.

A/J mice express a hypomorphic allele of NAIP5 (Diez et al., 2000), and A/J macrophages still activate caspase-1 in response to WT Lp under certain infection conditions (Lamkanfi et al., 2007). However, using *Naip5^−/−^* macrophages, it was shown that NAIP5 is required for caspase-1 activation in response to WT Lp (Lightfield et al., 2008). Interestingly, NAIP6 also interacts with *L. pneumophila* flagellin (Kofoed and Vance, 2011; Zhao et al., 2011). However, NAIP6 is insufficient for the restriction of *L. pneumophila*, as *Naip5^−/−^* macrophages and mice are permissive for infection (Lightfield et al., 2008), potentially due to lower expression levels of NAIP6 relative to NAIP5 in primary macrophages (Wright et al., 2003). NAIP5 and NLRC4 also contribute to the control of *L. pneumophila* replication by enhancing fusion of the *Legionella*-containing vacuole (LCV) with lysosomes during infections performed at a low multiplicity of infection (MOI) (Amer et al., 2006; Fortier et al., 2007). In addition, flagellin-dependent NLRC4 signaling leads to caspase-7-mediated restriction of *L. pneumophila* via enhanced lysosomal degradation of the bacterium (Akhter et al., 2009). NLRC4-mediated restriction *in vivo* is also partially caspase-1-independent through an unknown mechanism (Pereira et al., 2011). However, caspase-1 activation downstream of NLRC4 clearly induces pyroptosis and leads to IL-18 secretion both *in vitro* and *in vivo*, contributing to IFN-γ production and the subsequent resolution of pulmonary infection (Brieland et al., 2000; Spörri et al., 2006; Archer et al., 2009; Case et al., 2009). Thus, the NAIP5/NLRC4 inflammasome may control *L. pneumophila* replication through multiple mechanisms. Further studies are needed to determine the relative contributions of these mechanisms.

Not surprisingly, infection conditions, including MOI, can affect the detection of caspase-1 activation in response to *L. pneumophila*, as higher MOIs likely enhance the number of macrophages that harbor bacteria. At higher MOIs, infection of B6 macrophages induces both NLRC4-dependent and NLRC4-independent inflammasome activation. NLRC4-independent caspase-1 activation and IL-1β and IL-18 secretion require ASC and NLRP3, although the identity of the *L. pneumophila-*derived signal sensed via NLRP3 is unknown (Case et al., 2009, 2013; Casson et al., 2013). Caspase-1 cleavage in the absence of ASC can be detected in either the supernatant or the cytosol, depending on the MOI (Case et al., 2009; Abdelaziz et al., 2011a). ASC also drives formation of a punctate structure containing caspase-1 and NLRC4 in *L. pneumophila*-infected macrophages (Case and Roy, 2011). At early timepoints, pore formation is not observed in the absence of NLRC4, though cell death still occurs in the absence of ASC. Recruitment of NLRC4 into the ASC complex appears to dampen NLRC4 activity because pyroptosis occurs at a higher rate in the absence of ASC (Case and Roy, 2011). Further studies are needed to elucidate the temporal and spatial coordination of the ASC- and NLRC4-dependent inflammasomes and how they are triggered by *L. pneumophila*.

## Inflammasome activation in human cells

Unlike macrophages from most inbred mouse strains, human cells are permissive for *L. pneumophila* replication. The mechanisms underlying inflammasome-mediated control of *L. pneumophila* replication in human cells are unclear. Humans express only one homolog of the numerous murine NAIP paralogs (Scharf et al., 1996). The homolog, human NAIP (hNAIP), restricts growth of WT Lp (Vinzing et al., 2008). Additionally, the human NLRC4 homolog, human IPAF (hIPAF), also restricts *L. pneumophila* replication. Overexpression of full-length hNAIP in HEK293T cells increases cell death in response to *L. pneumophila* (Boniotto et al., 2012), and overexpression of hNAIP in the murine macrophage RAW264.7 cell line mediates flagellin-induced pyroptosis and IL-1β secretion (Katagiri et al., 2012), suggesting that it may function similarly to NAIP5. However, unlike NAIP5, hNAIP does not co-immunoprecipitate with flagellin and instead interacts with T3SS needle proteins (Zhao et al., 2011; Yang et al., 2013). Thus, it is unclear whether hNAIP senses flagellin or another *L. pneumophila-*derived ligand, how hNAIP restricts *L. pneumophila* replication, and if hNAIP contributes to cell death or IL-1β secretion in primary human cells.

**Figure 2:**
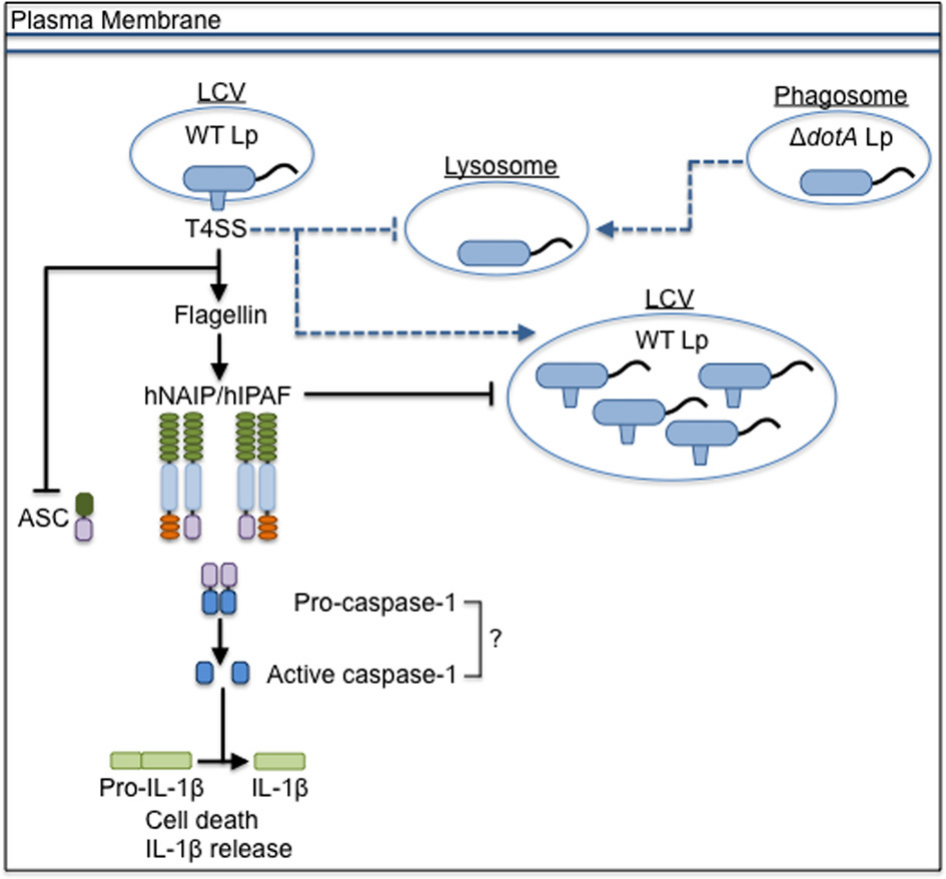
**The inflammasome-mediated response to *L. pneumophila* in human cells**. *L. pneumophila* that do not express a functional T4SS (Δ*dotA* Lp) traffic to the lysosome, but wild-type *L. pneumophila* (WT Lp) use the T4SS to translocate effectors into the host cytosol to establish a replicative niche, the *Legionella*-containing vacuole (LCV), and block fusion with lysosomes. The presence of flagellin triggers signaling through hNAIP/hIPAF that blocks replication of WT Lp, though it is unclear if caspase-1 is involved in restriction. T4SS activity down-regulates the expression of ASC. Dashed lines represent vesicular trafficking patterns. Solid lines represent pathways for activation of the host response. Arrows at the end of lines represent induction, while flat bars at the end of lines represent inhibition.

The implication that the IPAF/NAIP/caspase-1 inflammasome contributes to restriction of *L. pneumophila* is pervasive, though caspase-1 activation in response to *L. pneumophila* has not been explicitly shown in primary cells from humans, a naturally susceptible host. Immortalized human alveolar epithelial cells activate caspase-1 in response to *L. pneumophila*, though primary human monocytes and monocyte-derived macrophages (MDMs) do not produce detectable levels of processed or active caspase-1 (Santic et al., 2007; Furugen et al., 2008; Abdelaziz et al., 2011b). Additionally, the expression of ASC is moderately down-regulated in infected monocytes, potentially contributing to evasion of inflammasome activation in human cells by *L. pneumophila* (Abdelaziz et al., 2011b). Future studies in primary MDMs and human alveolar macrophages are needed to clarify the role of the inflammasome in restricting *L. pneumophila* replication in human cells (Figure 2).

## Inflammasome activation and autophagy

In murine macrophages, autophagy is induced shortly after phagocytosis of *L. pneumophila*, as components of the autophagy pathway co-localize with the LCV (Amer and Swanson, 2005). LCVs in A/J macrophages show delayed autophagosome maturation compared to LCVs in B6 macrophages, potentially contributing to increased replication of the bacterium. When expression of the autophagy component ATG5 is silenced, *L. pneumophila* replication in A/J macrophages increases. Additionally, replication of *L. pneumophila* decreases slightly when autophagy is induced exogenously, suggesting that autophagy contributes to restriction of *L. pneumophila* replication (Matsuda et al., 2009). Under low MOI infection conditions where there is minimal induction of pyroptosis, it was revealed that the induction of autophagy dampens pyroptosis in response to *L. pneumophila*, and turnover of autophagosomes requires NAIP5, NLRC4, and caspase-1 (Byrne et al., 2013). Collectively, these data suggest that NAIP5 inflammasome activation contributes to the restriction of *L. pneumophila* replication by inducing autophagy and/or pyroptosis, depending on the MOI and amount of flagellin present. How competing host and bacterial factors influence the outcome of inflammasome activation and autophagy during infection remains unclear and may be clarified by studies examining the temporal regulation of inflammasome activation and autophagy at a single-cell level.

## *L. pneumophila* and non-canonical inflammasome activation

Though Δ*flaA* Lp avoid NAIP5/NLRC4-mediated pyroptosis and can replicate in B6 macrophages, Δ*flaA* Lp trigger an additional form of cell death. Recently, caspase-11 has been shown to contribute to inflammasome activation in response to *L. pneumophila* (Figure 1). After MyD88 and TRIF-dependent upregulation of caspase-11, host cells undergo rapid caspase-11-mediated cell death, occurring in less than 4 h, in response to Δ*flaA* Lp (Case et al., 2013; Casson et al., 2013). Non-canonical inflammasome activation in response to Δ*flaA* Lp requires T4SS-mediated cytosolic access, as Δ*dotA* Lp do not activate caspase-11. Like caspase-1-mediated pyroptosis, caspase-11-dependent cell death leads to release of important inflammatory mediators, such as IL-1α, IL-1β, and IL-18. Caspase-11 is required for cell death and IL-1α release and additionally enhances NLRP3-dependent caspase-1 activation and IL-1β and IL-18 secretion (Case et al., 2013; Casson et al., 2013). IL-1α release *in vivo* is critical for host defense, including neutrophil recruitment to the airway space and control of bacterial burden, though there are caspase-11-independent sources of IL-1α *in vivo* as well (Barry et al., 2013; Casson et al., 2013). Caspase-11 also contributes to NAIP5/NLRC4-mediated inflammasome activation and restricts WT Lp by enhancing phago-lysosomal fusion (Akhter et al., 2012). In its non-lytic role, caspase-11 modulates actin polymerization and phosphorylation of cofilin to promote lysosomal trafficking of pathogenic, but not non-pathogenic, bacteria. Additionally, caspase-11 contributes to control of WT Lp replication *in vivo* (Akhter et al., 2012).

Caspase-11 responds not only to vacuolar bacteria that access the host cytosol through the T4SS but also to *L. pneumophila* that escape from the vacuole and aberrantly enter the cytosol (Aachoui et al., 2013). The T4SS-translocated effector SdhA is critical for bacterial growth in primary macrophages (Laguna et al., 2006; Liu et al., 2008). Macrophages infected with Δ*sdhA* Lp undergo cell death because SdhA is required to maintain LCV membrane integrity (Creasey and Isberg, 2012). Therefore, Δ*sdhA* Lp aberrantly enter the host cytosol where they become degraded, induce type I IFN, and activate caspase-1 via AIM2 (Monroe et al., 2009; Creasey and Isberg, 2012; Ge et al., 2012). In addition, Δ*sdhA* Lp induce rapid caspase-11-dependent cell death independently of bacterial flagellin (Aachoui et al., 2013). It appears that AIM2 responds to cytosolic *L. pneumophila* by producing IL-1β, whereas caspase-11 mediates cell death. However, *L. pneumophila* does not normally enter the cytosol, so the upstream mediators of caspase-11 activation may be different for Δ*sdhA*Δ*flaA* bacteria that enter the cytosol and Δ*flaA* bacteria that remain within the vacuole. Whether the bacteria physically enter the cytosol or not, these unique pathways upstream of caspase-11 are likely relevant for defense against other pathogens that lack or down-regulate flagellin during infection.

Non-canonical inflammasome activation is a recently described phenomenon, so there are many questions that remain unanswered. Currently, no NLRs have been identified that act upstream of caspase-11 to induce non-canonical inflammasome activation. As *L. pneumophila* rapidly and robustly activates caspase-11, it will be a valuable tool for future studies aiming to identify NLRs or other host factors critical for caspase-11 activation. The only bacterial factor that has been shown to initiate non-canonical inflammasome activation is cytosolic LPS (Hagar et al., 2013; Kayagaki et al., 2013). For some Gram-negative bacteria, it is thought that bacterial RNA may access the host cytosol to activate NLRP3 and caspase-11 (Kanneganti et al., 2006; Rathinam et al., 2012b). However, translocation of *L. pneumophila* RNA to initiate inflammasome activation has not been verified experimentally. Additionally, though cytosolic LPS may trigger caspase-11 during infection with Δ*sdhA* Lp that aberrantly enter the cytosol, it is unclear if LPS is sensed by host cells to initiate non-canonical inflammasome activation in the context of infection with *L. pneumophila* that remain within the LCV. Further studies are needed to clarify what triggers the host response to Δ*flaA* Lp and to elucidate the molecular pathways that lead to caspase-11-mediated cell death.

## Concluding remarks

Studying the inflammasome pathways triggered by the pathogen *L. pneumophila* has shaped our knowledge of how host cells are poised to respond to violation by intracellular pathogens. Whether the bacterium utilizes its T4SS to access the host cytosol, additionally delivers flagellin into the cytoplasm, or physically enters the cytosol itself, the host has evolved multiple ways to restrict replication of the pathogen and trigger immunity.

### Conflict of interest statement

The authors declare that the research was conducted in the absence of any commercial or financial relationships that could be construed as a potential conflict of interest.
